# Neutrophil to lymphocyte ratio as predictor for incident hypertension: a 9-year cohort study in Taiwan

**DOI:** 10.1038/s41440-019-0245-3

**Published:** 2019-03-08

**Authors:** Yi-Han Jhuang, Tung-Wei Kao, Tao-Chun Peng, Wei-Liang Chen, Yen-Wei Li, Pi-Kai Chang, Li-Wei Wu

**Affiliations:** 1 Division of Family Medicine, Department of Family and Community Medicine, Tri-Service General Hospital; and School of Medicine, National Defense Medical Center, Taipei, Taiwan Republic of China; 2 Division of Geriatric Medicine, Department of Family and Community Medicine, Tri-Service General Hospital; and School of Medicine, National Defense Medical Center, Taipei, Taiwan Republic of China; 3 Graduate Institute of Medical Sciences, National Defense Medical Center, Taipei, Taiwan Republic of China; 4 Division of Colon and Rectal Surgery, Department of Surgery, Tri-Service General Hospital; and School of Medicine, National Defense Medical Center, Taipei, Taiwan Republic of China

**Keywords:** Neutrophil-to-lymphocyte ratio (NLR), Hypertension, Taiwan

## Abstract

The neutrophil-to-lymphocyte ratio (NLR) has received much attention in recent decades and has been a novel inflammatory marker. NLR has been applied in predicting the prognosis of malignancies, mortality, and chronic diseases. Additionally, hypertension, defined as systolic blood pressure ≥ 140 mm Hg or diastolic blood pressure ≥ 90 mm Hg, a previous diagnosis of hypertension, and taking any antihypertensive drug, has been one of the most common chronic diseases in Asia and is currently the most important risk factor for cardiovascular diseases worldwide. Thus, we aimed to investigate the correlation between NLR and prevalent hypertension in the Taiwanese population. From the data of routine health checkups at the General Health Promotion Center in the Tri-Service General Hospital (TSGH), a total of 6278 participants were included. The NLR value was divided into tertiles. The Cox regression model revealed that the highest NLR group tended to be hypertensive (HR = 1.28, 95% CI 1.03–1.59) after adjustment. Individuals were also divided into age-specific, BMI-specific, or sex-specific groups; compared with the lowest NLR group, elderly individuals in the highest tertile of NLR were relatively likely to be hypertensive after covariate adjustment (HR = 1.88, 95% CI 1.19–2.96). Furthermore, a male group aged more than 60 years was reported to have a significant association with hypertension (HR = 1.84, 95% CI 1.06–3.18). However, there was no significant difference in the BMI-based stratified groups, even after adjustment. Our research revealed a significant association between the NLR and incident hypertension, especially in elderly or male Taiwanese individuals.

## Introduction

In previous studies, inflammatory and oxidative factors, such as C-reactive protein (CRP) and red blood cell distribution, have been thought to be related to cardiovascular disease [1, 2]. In contrast, we would like to find a relative cost-effect screening tool to screen individuals with chronic diseases, such as hypertension, type 2 diabetes, metabolic syndrome, etc. Upon admission, patients usually receive a series of biochemistry and blood profiles, including white blood cell (WBC) differential count. Moreover, the neutrophil-to-lymphocyte ratio (NLR) has received much attention in recent decades and has been a novel inflammatory and oxidative stress marker. The NLR comprises two immune pathways that are related to the oxidative response and the angiotensin II-mediated hypertensive response [3, 4]. The NLR has been applied in predicting the prognosis of malignancies, and patients with higher NLR had better outcomes than those without [5].

Hypertension is one of the most common chronic diseases in Asia and is currently the most important risk factor for cardiovascular diseases worldwide [6]. In addition to cardiovascular diseases, hypertension is also related to diabetes mellitus and even metabolic diseases [7]. In East Asian countries, such as Japan and China, hypertension has contributed to the increasing mortality of stroke, particularly compared with that of coronary heart disease (CHD) [8]. Therefore, the influence of hypertension was greater in Asian people than in Caucasian individuals. Perkovic et al. noted that the hazard ratio (HR) for stroke and CHD was higher for Asian than Caucasian populations, while systolic pressure increased 15 mm Hg [9]. In Taiwan, prehypertension (defined by systolic pressure between 120 and 139 mm Hg or diastolic pressure between 80 and 89 mm Hg) played an important role in the risk of cardiovascular disease [10]. Moreover, elderly people with obesity and metabolic syndrome tended to be more hypertensive than those without these conditions [10]. Many studies have noted the relationship between hypertension and NLR. Sun et al. demonstrated that a higher NLR in hypertensive individuals was associated with higher all-cause mortality after admission [11]. Moreover, NLR was higher in individuals with resistant hypertension than in those with normal blood pressure and controlled hypertension [12]. We also found that elevated NLR was significantly associated with prevalent hypertension in a large-scale epidemiological study in China [3]. However, unlike the previous study, we did not compare the kinds of markers, such NLR, platelet to lymphocyte ratio (PLR), and hypersensitive CRP, in predicting hypertension. However, we designed a 9-year cohort study in Taiwan to investigate the correlation between NLR and incident hypertension in individuals stratified by age, body mass index (BMI), and gender.

## Materials and methods

### Study design and participants

All data were collected from routine health checkups at the General Health Promotion Center in the TSGH, Taipei, Taiwan, from 2007 to 2015. A total of 52,644 participants in routine checkups were selected for this investigation. Information about medical history was collected through questionnaires by trained interviewers, including personal history of smoking. Participants received a series of physical examinations by physicians and anthropometric measurements by nurses. The exclusion criteria were determined and emphasized as follows. First, we excluded 2604 participants with a medical history of cardiovascular disease, hypertension, metabolic syndrome, and diabetes mellitus. Then, we excluded 543 participants with chronic and acute diseases, such as acute upper airway infection, or those taking medications that may affect the WBC concentration. Third, we excluded 43,219 participants without complete data or lost follow-up. Finally, a total of 6278 participants were enrolled in this study. The Institutional Review Board (IRB) of the TSGH approved the present study. The data were only used for medical research and subject to Helsinki Declaration and analyzed anonymously, so the IRB agreed to waive individual informed consent.

### Measurement of NLR

Blood sampling was performed for all participants; we excluded individuals with acute disease, which might affect the WBC and differential counts. The WBC and differential count were measured by using an Abbott Cell Dyn 3000 hematology analyzer (Abbott Laboratories, Abbott Park, IL, USA). Therefore, we obtained precise neutrophil and lymphocyte counts. The NLR was calculated as the neutrophil count ÷ lymphocyte count.

### Covariates

BMI represents the ratio of body mass tissue in a unit of kg/m^2^ and participants were asked to stand on a height–weight scale without shoes to record values. Blood pressure was typically measured on the participants’ right arm by mercury sphygmomanometer after the participants sat in rest for 5 min, unless any specific reason indicated that the procedure should not be done on the right arm. Blood pressure was measured three times, and the means were recorded.

Peripheral blood sampling for all participants was performed in the morning after fasting for at least 8 h. The blood specimens were collected in test tubes that may contain one or more additives, including the anticoagulant EDTA and heparin. Serum high-density lipoprotein cholesterol (HDL-C) and total cholesterol were measured by using the enzymatic cholesterol assay method with dextran sulfate precipitation. Serum uric acid was measured by using the Beckman Coulter AU5800 (Beckman Coulter Inc., Brea, CA, USA).

### Definition of hypertension

Hypertension was defined as systolic blood pressure ≥140 mm Hg or diastolic blood pressure ≥90 mm Hg, previous diagnosis of hypertension, and taking any antihypertensive drugs [13].

### Statistical analysis

We analyzed all data by IBM SPSS Statistics (IBM Corp. Released 2016. IBM SPSS Statistics for Mac, Version 24.0. Armonk, NY: IBM Corp.). Continuous data were presented in terms of the means with standard deviation, and categorical data were presented in terms of percentages. Two-sided *p*-values of <0.05 were regarded as indicating statistical significance. We used Student’s *t*-test to analyze continuous data and the Chi-square test to analyze categorical data in baseline characteristics. The NLR value was divided into tertiles: T1, <1.46; T2, 1.46–2.02; and T3, >2.02. The Cox regression model was utilized to determine the HRs of the tertiles of NLR for hypertension by comparing the higher tertile of NLR with the lowest tertile of NLR. Furthermore, the covariate in the baseline characteristics, which were thought to be potential confounding factors, was included in the extended model for adjustment. The covariates for adjustment were age, sex, BMI, serum WBC, serum total cholesterol, serum HDL-C, and smoking history. A stratified model was also conducted, and we classified our participants into age-specific, gender-specific, or BMI-specific groups separately. According to the World Health Organization, individuals older than 60 years were referred to as elderly persons in an age-specific group. In Taiwan, individuals whose BMI was larger than 27 were thought to be obese, those whose BMI was between 24 and 27 were defined as overweight, those whose BMI was between 18.5 and 24 were defined as healthy, and those whose BMI was <18.5 were defined as underweight according to the Health Promotion Administration, Ministry of Health and Welfare. Therefore, we conducted trend tests of different stratified layers to determine the further association between the HR of hypertension and the increasing tertiles of NLR.

## Results

Previous studies have indicated that compared with the lowest NLR group, the middle and highest tertiles of NLR seems to be related to hypertension [1]. In our study, we aimed to investigate the correlation between NLR and hypertension by age-, gender-, and BMI-specific groups. The clinical characteristics are shown in Table 1, the individuals in the highest tertile of NLR seem to be older and had higher levels of BMI, serum WBC, serum HDL-C, serum total cholesterol, and smoking history. To assess the relationship between NLR and hypertension, multivariable Cox regression analysis was used. Table 2 shows that the individuals in the highest tertile of NLR seemed to have hypertension compared to those in the lowest tertile of NLR after adjustment (HR = 1.28, 95% confidence interval (95% CI) 1.03–1.59), and this result was consistent with the previous study [14]. To further investigate the relationship between NLR and hypertension, we divided study individuals into groups on the basis of age, gender, and BMI, separately, as indicated in Table 2. In the BMI-specific and gender-specific groups, there was no significant difference, even after adjustment. However, elderly individuals in the highest tertile of NLR were 88% more likely to have hypertension those elderly individuals in the lowest NLR group (HR = 1.88, 95% CI 1.19–2.96), and we further investigated the male and female elderly group, which revealed a significant association between NLR and hypertension (HR = 1.84, 95% CI 1.06–3.18) after adjustment as shown in Table 3. In this study, we even investigated the correlation between the NLR and prehypertension and then divided the NLR into tertiles, but there was no statistical significance in age-, gender-, or BMI-specific group.

**Table 1:** Baseline characteristics of study group were categorized based on the tertile of neutrophil lymphocyte ratio and represented as means (SD) and proportion (number)

	Tertile of neutrophil lymphocyte ratio			*p* Value
	T1 (<1.46)	T2: (1.46–2.02)	T3 (≥2.02)	
Continuous variables				
Sample size (*N*)	2106	2065	2107	
Mean age	40.6 (12.8)	41.0 (12.6)	42.0 (12.9)	<0.01
BMI (kg/m^2^)	23.4 (3.6)	23.6 (3.7)	23.6 (3.9)	0.03
Systolic BP (mm Hg)	113.2 (17.1)	113.1 (18.3)	114.1 (26.8)	0.29
Diastolic BP (mm Hg)	72.0 (11.3)	72.3 (12.1)	72.3 (11.3)	0.70
Serum WBC (10^3^/μL)	5.75 (1.42)	6.12 (1.41)	6.94 (1.86)	<0.01
Serum HDL-cholesterol (mg/dL)	59.1 (16.4)	57.1 (16.0)	57.0 (15.9)	<0.01
Serum triglycerides (mg/dL)	106.2 (107.5)	111.7 (106.4)	106.9 (73.5)	0.17
Serum cholesterol (mg/dL)	187.5 (35.4)	187.3 (35.7)	184.0 (34.7)	<0.01
Serum BUN (mg/dL)	13.1 (3.5)	13.0 (3.5)	12.8 (4.4)	0.20
Serum creatinine (mg/dL)	0.8 (0.3)	0.8 (0.3)	0.8 (0.4)	0.10
Serum uric acid (mg/dL)	5.5 (1.5)	5.6 (2.3)	5.5 (1.5)	0.06
Categorical variables				
Male, % (*N*)	52.5 (1106)	51.0 (1053)	46.0 (969)	<0.01
History of smoking (*N*)	218	260	286	0.01

*SD* standard deviation, *BMI* body mass index, *BP* blood pressure, *WBC* white blood cell, *HDL* high-density lipoprotein, *BUN* blood urea nitrogen

**Table 2:** HRs of tertiles of neutrophil lymphocyte ratio for hypertension based on the age, gender, and BMI groups

Characteristics	Neutrophil lymphocyte ratios (tertiles)				*p* Value
	T1 (<1.46)	T2 (1.46–2.02)	*p* Value	T3 (≥2.02)	
	HR (95% CI)	HR (95% CI)		HR (95% CI)	
**Crude model**	1 (reference)	1.05 (0.84–1.30)	0.67	1.22 (0.99–1.51)	0.07
Age (years)					
<40	1 (reference)	1.18 (0.71–1.95)	0.53	0.88 (0.52–1.50)	0.65
40–60	1 (reference)	0.94 (0.70–1.26)	0.68	1.21 (0.91–1.61)	0.20
≥60	1 (reference)	1.38 (0.89–2.16)	0.15	2.05 (1.34–3.13)	<0.01
Gender					
Male	1 (reference)	1.04 (0.79–1.35)	0.80	1.34 (1.03–1.75)	0.03
Female	1 (reference)	1.09 (0.75–1.59)	0.66	1.21 (0.84–1.74)	0.30
Body mass index					
<18.5	1 (reference)	3.65 (0.40–33.38)	0.25	1.39 (0.09–22.86)	0.82
18.5–24	1 (reference)	1.00 (0.68–1.47)	0.99	1.23 (0.85–1.79)	0.27
24–27	1 (reference)	1.09 (0.74–1.60)	0.67	1.23 (0.84–1.80)	0.28
≥27	1 (reference)	0.99 (0.67–1.46)	0.96	1.04 (0.72–1.52)	0.83
**Extended model**	1 (reference)	1.13 (0.85–1.39)	0.35	1.28 (1.03–1.59)	0.03
Age (years)					
<40	1 (reference)	0.81 (0.48–1.37)	0.44	0.56 (0.31–0.99)	0.05
40–60	1 (reference)	1.01 (0.75–1.36)	0.96	1.31 (0.97–1.79)	0.08
≥60	1 (reference)	1.31 (0.83–2.07)	0.25	1.88 (1.19–2.96)	0.01
Gender					
Male	1 (reference)	1.02 (0.78–1.35)	0.86	1.24 (0.94–1.65)	0.13
Female	1 (reference)	1.24 (0.84–1.82)	0.27	1.17 (0.79–1.73)	0.43
Body mass index					
<18.5	1 (reference)	18.73 (0.47–744)	0.12	5.73 (0.04–905)	0.50
18.5–24	1 (reference)	0.88 (0.60–1.30)	0.53	1.11 (0.74–1.66)	0.63
24–27	1 (reference)	1.12 (0.75–1.69)	0.57	1.25 (0.83–1.88)	0.29
≥27	1 (reference)	1.18 (0.79–1.76)	0.42	1.21 (0.80–1.83)	0.38

Adjusted models were crude models adjusted for age, sex, BMI, serum WBC, serum total cholesterol, serum HDL-C, and smoking history

*HR* hazard ratio, *CI* confidence interval, *BMI* body mass index, *WBC* white blood cell, *HDL-C* high-density lipoprotein cholesterol

**Table 3:** HRs of tertiles of neutrophil lymphocyte ratio for hypertension based on the age- and gender-specific groups

Characteristics	Neutrophil lymphocyte ratios (tertiles)				*p* Value
	T1 (<1.46)	T2 (1.46–2.02)	*p* Value	T3 (≥2.02)	
	HR (95% CI)	HR (95% CI)		HR (95% CI)	
**Crude model**					
Male					
Age < 60	1 (reference)	0.94 (0.69–1.28)	0.69	1.13 (0.83–1.56)	0.44
Age ≥ 60	1 (reference)	1.47 (0.87–2.49)	0.15	2.09 (1.26–3.49)	0.01
Female					
Age < 60	1 (reference)	1.27 (0.83–1.95)	0.28	1.35 (0.89–2.05)	0.16
Age ≥ 60	1 (reference)	1.03 (0.42–2.53)	0.94	1.89 (0.83–4.30)	0.13
**Extended model**					
Male					
Age < 60	1 (reference)	0.92 (0.67–1.27)	0.62	1.08 (0.77–1.51)	0.65
Age ≥ 60	1 (reference)	1.34 (0.77–2.32)	0.30	1.84 (1.06–3.18)	0.03
Female					
Age < 60	1 (reference)	1.30 (0.84–2.01)	0.24	1.14 (0.73–1.78)	0.57
Age ≥ 60	1 (reference)	1.05 (0.42–2.67)	0.91	2.08 (0.87–4.97)	0.10

Adjusted models were crude models adjusted for age, sex, BMI, serum WBC, serum total cholesterol, serum HDL-C, and smoking history

*HR* hazard ratio, *CI* confidence interval, *BMI* body mass index, *WBC* white blood cell, *HDL-C* high-density lipoprotein cholesterol

## Discussions

Previous studies have documented that an elevated WBC count was relevant to an increased risk of hypertension in Japanese male office workers, and this relationship was also noted in nonsmokers, especially those who indicated WBC count as a marker of low-grade systemic inflammation to be a significant risk of hypertension [15]. Furthermore, the NLR played an important role in prevalent chronic diseases, and compared to the lowest level, the higher tertile of NLR was associated significantly with the likelihood of hypertension and diabetes mellitus [14], and a large-scale cohort study also demonstrated the same result, consistent with our study [3]. Forget et al. demonstrated that the normal range of NLR values is between 0.78 and 3.53 in a nongeriatric healthy population, but the complete documentation of smoking history and sex group was not been included in this study [16]. Similar to a previous study that presented a higher NLR as a novel and convenient predictor of all-cause mortality in hypertensive patients aged over 80 years, our research also revealed an association between NLR and incident hypertension, especially in elderly patients [11].

The mechanism underlying these correlations was explored. Neutrophils were thought to be mediators that regulate inflammatory processes involving complex interactions and are involved in releasing reactive oxygen species (ROS). Inflammation may affect the level of nitric oxide (NO), which may cause vascular endothelium injury [3] potentially associated with hypertension [17, 18]. Additionally, NO is a marker of oxidative stress. ROS are involved in NO-based cell signaling and can cause vascular disorders by compromising NO function [19]. Ghosh et al. also demonstrated that oxidative stress contributed to prevalent hypertension in an animal model [20]. In addition, oxidative stress and endothelial dysfunction were relevant to atherogenesis [21].

T lymphocytes, known as lymphocyte subsets, participate in the hypertensive process. The control of blood pressure was also related to antigen-driven autoimmune regulation; one of the main theories was that T lymphocytes were activated by T-cell receptor ligation and a costimulatory interaction between CD28 (presented on T lymphocytes) and B7 ligation (presented on antigen presenting cells). The blockage or limitation of these two pathways attenuated the hypertensive condition [22, 23]. Furthermore, the NADPH subunit p47^phox^ on T lymphocytes also played an important role in the angiotensin II-mediated hypertensive response. Additionally, T lymphocytes also infiltrated organs (including vascular, heart, and kidney), which may lead to aortic stiffness and endothelial dysfunction and further enhance hypertension [4]. Crowley et al. found that T lymphocytes infiltrated the renal interstitium, especially around the renal vasculature [24]. In Korea, Youn et al. demonstrated that increased CD8+ T cells and enhanced CXC chemokine receptor type 3 were noted in human hypertension [25]. However, the relationship between T lymphocytes and hypertension in humans requires further investigation.

To our knowledge, this study is the first to utilize the NLR as an inflammatory marker and investigate the association between the NLR and hypertension stratified by sex, age, and BMI in Taiwan. Our research also provided a large number of participants with a 9-year follow-up and the framework that the NLR might be a novel, approachable, and cost-effective inflammatory marker, especially in male and elderly groups.

However, there were some limitations. First, unmeasured confounding factors might exist, even though we tried hard to select against these factors. Fortunately, the enrolled individuals were relatively healthy, so we could minimize the effect of unknown confounders. Second, individuals with higher but normal levels of NLR are more likely to visit a doctor, and bias could occur, so hypertension might be found by chance. However, our research enrolled a large number of participants and decreased the proportion of individuals who visited doctor, so that we might eliminate this effect. Third, a number of participants were excluded due to incomplete data or lost follow-up; therefore, selection bias was noted in our study. Although there were some limitations, our study might elucidate the role of NLR-associated hypertension and relevant underlying mechanisms, primarily in male and elderly individuals. Further work is needed to provide stronger evidence in NLR in relation to hypertension.

In our study, we documented that NLR played an important role in incident hypertension. After stratifying the participants, we found that male and elderly individuals in the higher NLR group tended to be more hypertensive than those in the lowest NLR group. However, there was no statistically significant difference in the female, younger, and BMI-specific groups. Therefore, in the clinic, we might use NLR as an index for hypertension, especially in both the male and elderly groups in Taiwan.
